#  Persistent Racial/Ethnic Disparities in Fatal Unintentional Drowning Rates Among Persons Aged ≤29 Years — United States, 1999–2019

**DOI:** 10.15585/mmwr.mm7024a1

**Published:** 2021-06-18

**Authors:** Tessa Clemens, Briana Moreland, Robin Lee

**Affiliations:** ^1^Division of Injury Prevention, National Center for Injury Prevention and Control, CDC; ^2^Synergy America Inc. Duluth, Georgia.

During 1999–2019, a total of 81,947 unintentional drowning deaths occurred in the United States (*1*). Drowning is one of the three leading causes of unintentional injury death among persons aged ≤29 years and results in more deaths among children aged 1–4 years than any other cause except birth defects (*2*). Drowning death rates have decreased since 1990 (declining by 57% worldwide and by 32% in the United States) (*3*). However, because of racial/ethnic disparities in drowning risk, rates remain high among certain racial/ethnic groups, particularly American Indian or Alaska Native (AI/AN) persons and Black or African-American (Black) persons (*4*). To assess whether decreasing drowning death rates have been accompanied by reductions in racial/ethnic disparities, and to further describe these disparities by age group and setting, CDC analyzed U.S. mortality data during 1999–2019. The drowning death rate among persons aged ≤29 years was 1.3 per 100,000 population. The rate per 100,000 among AI/AN persons (2.5) and Black persons (1.8) was higher than among all other racial/ethnic groups and was 2.0 and 1.5 times higher than among White persons (1.2). Racial/ethnic disparities in drowning death rates did not significantly decline for most groups, and the disparity in rates among Black persons compared with White persons increased significantly from 2005–2019. Drowning death rates are associated with persistent and concerning racial/ethnic disparities. A better understanding of the factors that contribute to drowning disparities is needed. Implementing and evaluating community-based interventions, including those promoting basic swimming and water safety skills, among disproportionately affected racial/ethnic groups could help reduce drowning disparities.

National Vital Statistics System death certificate data from 1999–2019 were used to calculate unintentional drowning death rates and disparity rate ratios (RRs) for persons aged ≤29 years. Crude death rates (per 100,000 population) were calculated using 1999–2019 U.S. Census bridged-race population estimates. Disparity RRs and their corresponding 95% confidence intervals (CIs) were calculated using White persons as the reference population (chosen because they represented the largest racial/ethnic group during the study period). RRs >1.0 indicate a higher drowning death rate in the specified group compared with White persons. Because of high interannual variability in drowning death rates, 5-year moving averages in rates and RRs were calculated to visualize temporal trends.

Unintentional drowning deaths were identified using the *International Classification of Diseases, Tenth Revision* underlying cause of death codes W65–W74, V90, and V92. Death rates and RRs were examined by setting (bathtub, swimming pool, natural water, watercraft, and other or unspecified), age, and race/ethnicity. Race/ethnicity was categorized as non-Hispanic AI/AN, non-Hispanic Asian or Pacific Islander (A/PI), non-Hispanic Black, non-Hispanic White, and Hispanic or Latino (referred to as Hispanic in this report). Age was categorized in 5-year age groups except for infants aged <1 year. Joinpoint regression (version 4.7.0.0; National Cancer Institute) was used to describe trends and changes in trends in annual drowning death rates and RRs. Up to three changes in trend could be detected. P-values <0.05 were considered significant. This activity was reviewed by CDC and was conducted consistent with applicable federal law and CDC policy.*

During 1999–2019, a total of 34,315 persons aged ≤29 years died from unintentional drowning in the United States (Table 1). The 5-year moving average in crude drowning death rates decreased from 1.5 to 1.2 per 100,000 population during the study period (Figure). From 1999 to 2019, annual rates significantly decreased for each racial/ethnic group except AI/AN (p = 0.16) and Hispanic persons (p = 0.29). The highest annual drowning death rates occurred among AI/AN (range: 1.8–3.6) and Black (range: 1.6–2.5) persons. Using White persons as the reference, the 5-year moving average in drowning RRs ranged from 1.8 to 2.2 for AI/AN persons and from 1.3 to 1.6 for Black persons (Figure). The Black:White RR decreased significantly from 1999 to 2005 (p = 0.04) and then increased significantly from 2005 to 2019 (p = 0.003). There was no significant change in the AI/AN:White (p = 0.16) or A/PI:White (p = 0.15) RRs during 1999–2019. The Hispanic:White RR decreased significantly from 1999 to 2015 (p<0.001) and did not change significantly from 2015 to 2019 (p = 0.19).

**TABLE 1:** Numbers and rates* of fatal unintentional drowning^†^ among persons aged ≤29 years, by age group, setting, and race/ethnicity — United States, 1999–2019

Setting	Age group, yrs															
	<1		1–4		5–9		10–14		15–19		20–24		25–29		Total	
	No.	Rate	No.	Rate	No.	Rate	No.	Rate	No.	Rate	No.	Rate	No.	Rate	No.	Rate
**All settings**																
AI/AN	24	2.8	136	4.1	52	1.2	44	1.0	108	2.3	136	3.1	154	3.9	**654**	**2.5**
A/PI	23	0.5	236	1.4	164	0.8	117	0.6	326	1.5	325	1.3	322	1.1	**1,513**	**1.1**
Black	224	1.8	1,205	2.4	976	1.5	1,041	1.6	1,615	2.4	1,198	1.8	919	1.5	**7,178**	**1.8**
Hispanic^§^	236	1.2	1,707	2.2	428	0.5	384	0.4	1,126	1.3	1,424	1.6	1,066	1.2	**6,371**	**1.2**
White	516	1.2	5,964	3.3	1,392	0.6	1,083	0.4	3,103	1.2	3,485	1.3	2,943	1.1	**18,486**	**1.2**
Total**	1,028	1.2	9,269	2.8	3,025	0.7	2,679	0.6	6,295	1.4	6,590	1.5	5,429	1.2	**34,315**	**1.3**
**Pool**																
AI/AN	—^¶^	—	36	1.1	13	—	—	—	—	—	—	—	—	—	**62**	**0.2**
A/PI	—	—	132	0.8	67	0.3	26	0.1	37	0.2	37	0.2	66	0.2	**369**	**0.3**
Black	—	—	697	1.4	490	0.8	302	0.5	261	0.4	171	0.3	106	0.2	**2,035**	**0.5**
Hispanic^§^	12	—	952	1.2	157	0.2	74	<0.1	101	0.1	116	0.1	94	0.1	**1,506**	**0.3**
White	61	0.1	3,165	1.7	414	0.2	149	<0.1	180	<0.1	189	<0.1	185	<0.1	**4,343**	**0.3**
Total**	86	0.1	4,996	1.5	1,148	0.3	560	0.1	581	0.1	532	0.1	453	0.1	**8,347**	**0.3**
**Natural water**																
AI/AN	—	—	50	1.5	24	0.6	27	0.6	62	1.3	75	1.7	81	2.1	**319**	**1.2**
A/PI	—	—	48	0.3	61	0.3	70	0.3	214	1.0	203	0.8	188	0.7	**785**	**0.6**
Black	—	—	162	0.3	241	0.4	420	0.6	879	1.3	649	1.0	464	0.8	**2,817**	**0.7**
Hispanic^§^	—	—	224	0.3	148	0.2	208	0.2	696	0.8	865	1.0	629	0.7	**2,775**	**0.5**
White	14	—	1,055	0.6	495	0.2	470	0.2	1,684	0.6	1,796	0.7	1,354	0.5	**6,868**	**0.5**
Total**	23	<0.1	1,540	0.5	973	0.2	1,199	0.3	3,542	0.8	3,600	0.8	2,727	0.6	**13,604**	**0.5**
**Watercraft**																
AI/AN	—	—	—	—	—	—	—	—	13	—	19	—	26	0.7	**66**	**0.3**
A/PI	—	—	—	—	—	—	—	—	17	—	24	<0.1	16	—	**62**	**<0.1**
Black	—	—	—	—	13	—	19	—	61	<0.1	70	0.1	72	0.1	**239**	**<0.1**
Hispanic^§^	—	—	—	—	—	—	15	—	37	<0.0	85	<0.1	73	<0.1	**228**	**<0.1**
White	—	—	41	<0.1	73	<0.1	102	<0.1	307	0.1	484	0.2	426	0.2	**1,438**	**<0.1**
Total**	—	—	56	<0.1	98	<0.1	143	<0.1	436	<0.1	682	0.2	617	0.1	**2,038**	**<0.1**
**Bathtub**																
AI/AN	17	—	10	—	—	—	—	—	—	—	—	—	—	—	**39**	**0.2**
A/PI	13	—	15	—	—	—	—	—	—	—	—	—	—	–	**51**	**<0.1**
Black	151	1.2	129	0.3	29	<0.1	34	<0.1	23	<0.1	43	<0.1	55	<0.1	**464**	**0.1**
Hispanic^§^	157	0.8	182	0.2	21	<0.1	22	<0.1	30	<0.1	30	<0.1	38	<0.1	**480**	**<0.1**
White	319	0.7	471	0.3	85	<0.1	111	<0.1	151	<0.1	256	<0.1	324	0.1	**1,717**	**0.1**
Total**	658	0.8	811	0.2	139	<0.1	169	<0.1	207	<0.1	339	<0.1	434	<0.1	**2,757**	**0.1**
**Other or unspecified**																
AI/AN	—	—	39	1.2	13	—	—	—	31	0.7	34	0.8	36	0.9	**168**	**0.7**
A/PI	—	—	39	0.2	31	0.2	17	—	56	0.3	53	0.2	45	0.2	**246**	**0.2**
Black	62	0.5	214	0.4	203	0.3	266	0.4	391	0.6	265	0.4	222	0.4	**1,623**	**0.4**
Hispanic^§^	62	0.3	340	0.4	93	<0.1	65	<0.1	262	0.3	328	0.4	232	0.3	**1,382**	**0.3**
White	117	0.3	1,232	0.7	325	0.1	251	<0.1	781	0.3	760	0.3	654	0.3	**4,120**	**0.3**
Total**	255	0.3	1,866	0.6	667	0.2	608	0.1	1,529	0.3	1,446	0.3	1,198	0.3	**7,569**	**0.3**

**Abbreviations:** AI/AN = American Indian or Alaska Native; A/PI = Asian or Pacific Islander; ICD-10 = *International Classification of Diseases, Tenth Revision.* *Per 100,000 population. ^† ^ ICD-10 underlying cause of death codes W65–W74, V90, and V92. ^§^ Persons identified as Hispanic might be of any race. Persons identified as AI/AN, A/PI, Black, or White are all non-Hispanic. ^¶^ Dashes indicate death counts based on <10 deaths suppressed for confidentiality; death rates based on <20 deaths suppressed for unreliability.  ** Total rates for each setting include race/ethnicity “not stated.”

**FIGURE:**
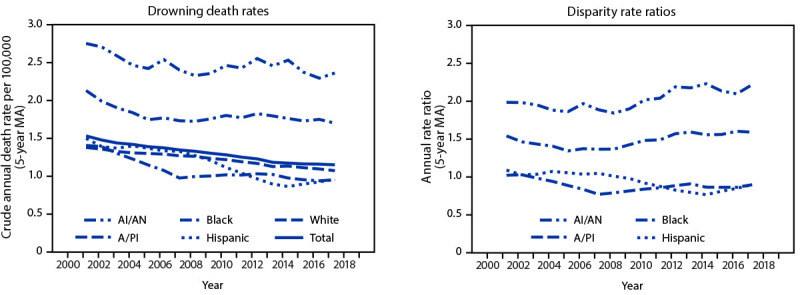
Five-year moving average* fatal unintentional drowning^†^ rates and rate ratios^§^ among persons aged ≤29 years, by race/ethnicity^¶^ — United States, 1999–2019 **Abbreviations:** AI/AN = American Indian or Alaska Native; A/PI = Asian or Pacific Islander; ICD-10 = *International Classification of Diseases, Tenth Revision*; MA = moving average. * Because of high interannual variability in drowning rates, 5-year MAs in rates and rate ratios were calculated to visualize temporal trends; annual rates and rate ratios are reported in text. For the study period (1999–2019), the first year for which a 5-year average can be calculated is 2001, and the last year for which a 5-year average can be calculated is 2017.  ^†^ ICD-10 underlying cause of death codes W65–W74, V90, and V92.  ^§^ Rate ratios use White persons as the comparison group.  ^¶^ Persons identified as Hispanic might be of any race. Persons identified in the other categories (AI/AN, A/PI, Black, or White) are all non-Hispanic.

Compared with the drowning death rate overall (all settings, ages, and years combined) among White persons, the rate was 2.0 times higher among AI/AN persons and 1.5 times higher among Black persons (Table 2); rates were lower among Hispanic (RR = 0.9) and A/PI persons (RR = 0.9). Drowning death rates and RRs varied by age and setting. For all settings combined, disparities in AI/AN rates were present across all age groups: the highest RRs were among persons aged 25–29 years (3.5), followed by children aged <1 year (2.5). Disparities in drowning death rates between Black persons and White persons were present across all age groups except persons aged 1–4 years, the largest being among children aged 10–14 years (RR = 3.6) and 5–9 years (RR = 2.6).

**TABLE 2:** Fatal unintentional drowning* disparity rate ratio among persons aged ≤29 years, by age group, setting, and race/ethnicity — United States, 1999–2019

Setting	Age group, yrs							
	<1	1–4	5–9	10–14	15–19	20–24	25–29	Total
	RR (95% CI)	RR (95% CI)	RR (95% CI)	RR (95% CI)	RR (95% CI)	RR (95% CI)	RR (95% CI)	RR (95% CI)
**All settings**								
AI/AN	2.5 (1.6–3.7)	1.2 (1.1–1.5)	2.1 (1.6–2.7)	2.3 (1.7–3.0)	2.0 (1.6–2.4)	2.4 (2.0–2.8)	3.5 (2.9–4.1)	**2.0 (1.9–2.2)**
A/PI	0.5 (0.3–0.7)	0.4 (0.4–0.5)	1.3 (1.1–1.6)	1.3 (1.1–1.6)	1.3 (1.1–1.4)	1.0 (0.9–1.1)	1.0 (0.9–1.1)	**0.9 (0.8–0.9)**
Black	1.6 (1.3–1.8)	0.7 (0.7–0.8)	2.6 (2.4–2.8)	3.6 (3.3–3.9)	2.0 (1.9–2.1)	1.4 (1.3–1.5)	1.3 (1.2–1.4)	**1.5 (1.5–1.5)**
Hispanic^†^	1.0 (0.9–1.2)	0.7 (0.6–0.7)	0.8 (0.7–0.9)	1.0 (0.9–1.1)	1.1 (1.0–1.2)	1.2 (1.1–1.3)	1.1 (1.0–1.2)	**0.9 (0.9–1.0)**
White	Ref	Ref	Ref	Ref	Ref	Ref	Ref	**Ref**
**Pool**								
AI/AN	—^§^	0.6 (0.5–0.9)	—	—	—	—	—	**0.8 (0.6–1.1)**
A/PI	—	0.4 (0.4–0.5)	1.8 (1.4–2.4)	2.1 (1.4–3.2)	2.5 (1.7–3.5)	2.0 (1.4–2.9)	3.2 (2.4–4.2)	**0.9 (0.8–1.0)**
Black	—	0.8 (0.7–0.9)	4.4 (3.8–5.0)	7.6 (6.3–9.3)	5.6 (4.6–6.7)	3.6 (2.9–4.4)	2.4 (1.9–3.1)	**1.8 (1.7–1.9)**
Hispanic^†^	—	0.7 (0.6–0.7)	1.0 (0.8–1.1)	1.4 (1.0–1.8)	1.7 (1.3–2.1)	1.8 (1.4–2.3)	1.5 (1.2–1.9)	**1.0 (0.9–1.0)**
White	Ref	Ref	Ref	Ref	Ref	Ref	Ref	Ref
**Natural water**								
AI/AN	—	2.6 (1.9–3.4)	2.7 (1.8–4.0)	3.2 (2.2–4.7)	2.1 (1.6–2.7)	2.5 (2.0–3.2)	4.0 (3.2–5.0)	**2.7 (2.4–3.0)**
A/PI	—	0.5 (0.4–0.7)	1.4 (1.1–1.8)	1.8 (1.4–2.3)	1.5 (1.3–1.8)	1.2 (1.0–1.4)	1.2 (1.1–1.4)	**1.2 (1.1–1.3)**
Black	—	0.6 (0.5–0.7)	1.8 (1.5–2.1)	3.4 (2.9–3.8)	2.0 (1.9–2.2)	1.4 (1.3–1.6)	1.5 (1.3–1.6)	**1.6 (1.5–1.7)**
Hispanic^†^	—	0.5 (0.4–0.6)	0.8 (0.6–0.9)	1.2 (1.0–1.5)	1.2 (1.1–1.4)	1.4 (1.3–1.6)	1.4 (1.3–1.5)	**1.1 (1.1–1.2)**
White	Ref	Ref	Ref	Ref	Ref	Ref	Ref	**Ref**
**Watercraft**								
AI/AN	—	—	—	—	—	—	4.1 (2.7–6.0)	**2.7 (2.1–3.4)**
A/PI	—	—	—	—	—	0.5 (0.3–0.8)	—	**0.5 (0.4–0.6)**
Black	—	—	—	—	0.8 (0.6–1.0)	0.6 (0.5–0.7)	0.7 (0.6–0.9)	**0.6 (0.6–0.7)**
Hispanic^†^	—	—	—	—	0.4 (0.3–0.5)	0.5 (0.4–0.7)	0.5 (0.4–0.7)	**0.4 (0.4–0.5)**
White	Ref	Ref	Ref	Ref	Ref	Ref	Ref	**Ref**
**Bathtub**								
AI/AN	—	—	—	—	—	—	—	**1.3 (1.0–1.8)**
A/PI	—	—	—	—	—	—	—	**0.3 (0.2–0.4)**
Black	1.7 (1.4–2.1)	1.0 (0.8–1.2)	1.3 (0.8–1.9)	1.2 (0.8–1.7)	0.6 (0.4–0.9)	0.7 (0.5–0.9)	0.7 (0.5–1.0)	**1.0 (0.9–1.2)**
Hispanic^†^	1.1 (0.9–1.3)	0.9 (0.8–1.0)	0.6 (0.4–1.0)	0.6 (0.4–0.9)	0.6 (0.4–0.9)	0.4 (0.2–0.5)	0.4 (0.3–0.5)	**0.8 (0.7–0.9)**
White	Ref	Ref	Ref	Ref	Ref	Ref	Ref	**Ref**
**Other or unspecified**								
AI/AN	—	1.7 (1.3–2.4)	—	—	2.2 (1.6–3.2)	2.7 (1.9–3.8)	3.6 (2.6–5.1)	**2.4 (2.0–2.8)**
A/PI	—	0.3 (0.2–0.5)	1.1 (0.7–1.6)	—	0.9 (0.7–1.1)	0.7 (0.6–1.0)	0.6 (0.5–0.8)	**0.6 (0.6–0.7)**
Black	1.9 (1.4–2.6)	0.6 (0.5–0.7)	2.3 (1.9–2.7)	3.9 (3.4–4.7)	1.9 (1.7–2.2)	1.4 (1.2–1.6)	1.5 (1.2–1.7)	**1.5 (1.4–1.6)**
Hispanic^†^	1.2 (0.9–1.6)	0.6 (0.6–0.7)	0.7 (0.6–0.9)	0.7 (0.6–1.0)	1.0 (0.9–1.2)	1.3 (1.1–1.5)	1.1 (0.9–1.2)	**0.9 (0.9–1.0)**
White	Ref	Ref	Ref	Ref	Ref	Ref	Ref	**Ref**

**Abbreviations:** AI/AN = American Indian or Alaska Native; A/PI = Asian or Pacific Islander; CI = confidence interval; ICD-10 = *International Classification of Diseases, Tenth Revision*; Ref = reference; RR = rate ratio. * ICD-10 underlying cause of death codes W65–W74, V90, and V92. ^†^ Persons identified as Hispanic might be of any race. Persons identified as AI/AN, A/PI, Black, or White are all non-Hispanic.  ^§^ Dashes indicate RRs based on <20 deaths suppressed for unreliability.

Racial/ethnic disparities were present in all settings and were most pronounced in swimming pool deaths; compared with White persons, the highest RRs occurred among Black youth aged 10–14 years (7.6), 15–19 years (5.6), and 5–9 years (4.4) (Table 2). Disparities in swimming pool drowning death rates were also present in most age groups for A/PI and Hispanic persons, with the highest RRs observed among those aged 25–29 years (3.2), 15–19 years (2.5), and 10–14 years (2.1) for A/PI persons and among those aged 20–24 years (1.8), 15–19 years (1.7), and 25–29 years (1.5) for Hispanic persons. Drowning death rates in natural water were highest among AI/AN persons (RR = 2.7), with high RRs across all age groups (range: 2.1–4.0). The drowning death rate in natural water among Black persons was 1.6 times higher than among White persons, with the highest RR found among children aged 10–14 years (3.4).

## Discussion

Racial/ethnic disparities in unintentional drowning death rates among persons aged ≤29 years were evident in 1999 and persisted through 2019, with significantly higher rates among AI/AN and Black persons compared with White, A/PI, and Hispanic persons. Although drowning death rates decreased overall, racial/ethnic disparities persisted during the 21-year period, and the disparity between Black and White persons increased in recent years.

Multiple factors contribute to increased risk of drowning for all persons, including behavior, skill (e.g., low water competency^ꝉ^), environment, and underlying medical conditions (*5*). Racial/ethnic differences in drowning death rates might reflect variation in these or other social or cultural factors among groups. Relying on death certificates to describe drowning disparities limits the ability to explore these factors, because death certificates do not include details on known risk or protective factors (*4*) or other sociocultural influences. Further research is needed on the determinants that contribute to racial/ethnic disparities in drowning, including the barriers to implementing effective drowning prevention programs in communities at highest risk.

Proven drowning prevention strategies include installing barriers that prevent unintended access to water, teaching basic swimming and water safety skills, using life jackets properly, active supervision, and knowing and performing cardiopulmonary resuscitation (CPR) (*4*). Racial/ethnic disparities in drowning deaths differed by setting, and the most applicable drowning prevention strategies might also differ by setting; however, having basic swimming and water safety skills can be beneficial in all settings (*4*). Research suggests that Black persons report more limited swimming ability than members of other groups (*6*,*7*). This disparity in swimming ability has persisted over time (*8*). Racial differences in fear of drowning have been identified as one factor contributing to limited swimming ability in some Black youths (*9*). A reduction in Black:White drowning disparities occurred in Florida from 1970 to 2015 (*10*). This progress might be the result of community-level initiatives to promote swimming skills among Black children (*10*). Swimming skill and other factors contributing to increased drowning risk in AI/AN persons have not been thoroughly explored. Engagement of the populations and communities at highest risk of drowning is critical to developing effective programs and reducing disparities.

The findings in this report are subject to at least three limitations. First, information about race/ethnicity on death certificates is reported by next of kin or by observation. Persons who self-report their race/ethnicity as AI/AN, Asian, or Hispanic are sometimes reported as White or non-Hispanic on death certificates, leading to possible underestimations of deaths among these groups; proxy reporting of race/ethnicity is especially inaccurate for AI/AN persons (*1*). Second, approximately 17% of drowning deaths were coded as “unspecified drowning,” meaning the setting could not be determined, and the drowning might have occurred in one of the other settings. Finally, because of a lack of exposure data, how the drowning disparities reported by setting are affected by a group’s exposure to that setting could not be determined.

Drowning is preventable, and more prevention efforts are needed to reduce the racial/ethnic disparities in drowning death rates that persist in the United States. Identification and evaluation of factors contributing to racial/ethnic disparities are crucial to inform the development and implementation of interventions that could effectively reduce disparities. Developing, implementing, and evaluating community-based interventions to promote drowning prevention strategies (installing barriers, basic swimming and water safety skills, using life jackets properly, active supervision, and knowing/performing CPR) among disproportionately affected racial/ethnic groups could help reduce disparities. Although the practicality of prevention strategies varies by setting, having basic swimming and water safety skills is applicable in all settings. Engaging populations at the highest risk of drowning to understand and address the barriers to accessing basic swimming and water safety skills training is needed.

**Summary**

**What is already known about this topic?**

Drowning is preventable; however, it is one of three leading causes of unintentional injury death among persons aged ≤29 years.

**What is added by this report?**

During 1999–2019, 34,315 persons aged ≤29 years died from drowning in the United States, and drowning death rates decreased from 1.5 to 1.2 per 100,000 population overall. Compared with non-Hispanic White persons, the rate was 2.0 times higher among American Indian or Alaska Native persons and 1.5 times higher among non-Hispanic Black persons. Disparities in drowning death rates between non-Hispanic Black and White persons increased from 2005 to 2019.

**What are the implications for public health practice?**

Although drowning death rates have decreased overall, racial/ethnic disparities persist. Implementing and evaluating community-based interventions, including those promoting basic swimming and water safety skills among disproportionately affected racial/ethnic groups, could help reduce these disparities.

## Footnotes

* 45 C.F.R. part 46.102(l)(2), 21 C.F.R. part 56; 42 U.S.C. Sect. 241(d); 5 U.S.C. Sect. 552a; 44 U.S.C. Sect. 3501 et seq.
